# Standard expected years of life lost (SEYLL) due to chronic obstructive pulmonary disease (COPD) in Poland from 1999 to 2014

**DOI:** 10.1371/journal.pone.0213581

**Published:** 2019-03-12

**Authors:** Elzbieta Dziankowska-Zaborszczyk, Marek Bryla, Beata Ciabiada-Bryla, Irena Maniecka-Bryla

**Affiliations:** 1 Department of Epidemiology and Biostatistics, Medical University of Lodz, Lodz, Poland; 2 Department of Social Medicine, Medical University of Lodz, Lodz, Poland; 3 Department of Preventive Medicine, Medical University of Lodz, Lodz, Poland; Sciensano, BELGIUM

## Abstract

**Purpose:**

The aim of the study is to analyze the standard expected years of life lost (SEYLL) due to chronic obstructive pulmonary disease (COPD) in Poland from 1999 to 2014 by sex and place of residence.

**Methods:**

The number of deaths due to chronic obstructive pulmonary disease (J40 –J44 and J47 according to ICD-10) over the period 1999 to 2014 was analyzed based on data obtained from the Central Statistical Office in Poland. Standard expected years of life lost due to chronic obstructive pulmonary disease were calculated by sex and place of residence according to the living population (SEYLL_p_) and the number of deaths caused by the disease (SEYLL_d_). Changes in the calculated measures were evaluated using joinpoint models. The annual percentage change (APC) and the average annual percentage change (AAPC) were also calculated.

**Results:**

The study revealed that COPD contributed to 1.8% of the total number of deaths which occurred between 1999 and 2014. The greatest decrease in the analyzed measures was observed among males from rural areas (p<0.05) (SEYLL: AAPC = -1.6; 95%CI: -3.0;-0.2; SEYLL_p_: AAPC = -2.0; 95%CI: -3.4;-0.6; SEYLL_d_: AAPC = -1.1; 95%CI: -1.2;-0.9). A statistically significant increase in the SEYLL and SEYLL_p_ indices was observed among female city dwellers (SEYLL: AAPC = 2.4; 95%CI:0.7;4.0 and SEYLL_p_: AAPC = 2.4; 95%CI: 0.8;4.1).

**Conclusions:**

All studied measures were higher in the male group than in the female group, regardless of the place of residence. A male who died of COPD in Poland in 2014 potentially lost 14.9 years of life, whereas a female lost 14.2 years.

## Introduction

Health inequalities and the need to eliminate them by improving the overall level of health of a population represent global challenges for public health. This can be done by reducing mortality rates and increasing life expectancy, as increased average life expectancy is mainly a consequence of decreased mortality risks due to the most significant causes of death.

The mortality rate depends on many factors, among which lifestyle, the environment and quality of medical care play a great role. These factors, in turn, are closely related to the socioeconomic status of a particular population. In the period of systemic transformation, the education level increased, which resulted in improved prophylaxis and more effective treatment of risk-factor induced diseases. Simultaneously, due to allocation of increased amount of money to the health care system, modern diagnostic and therapeutic methods become more available. In the light of these positive trends, pro-health programs, aimed at eliminating the most serious non-communicable diseases, are implemented. Screening tests are more commonly conducted. They enable to identify risk factors and early stages of a disease. All these translate into longer life expectancy of citizens of Poland [1].

In 1999, male life expectancy in Poland was 68.8 and it reached 73.8 years in 2014. Female life expectancy increased from 77.5 in 1999 to 81.6 in 2014. Recently, the dynamics of life expectancy extension in Poland slowed down; in 2017, male life expectancy was 74.0, and female– 81.8 years [2].

It is estimated that ischemic heart disease, cerebral stroke and COPD contribute to almost 32% of all deaths worldwide [3]; however, while mortality due to cardiovascular diseases and cerebral stroke has been decreasing in Europe in recent years, the number of deaths of COPD has been increasing [4]. Unfortunately, there is little awareness of this disease among the Polish population. A random survey conducted among Poles aged 15 and above in 2013 found that only 3% had heard the term *COPD* and understood what it meant, while 11% had heard it but did not know what it referred to. Half of those surveyed were not aware of methods of preventing COPD. More worryingly, only 25% of those affected by COPD were aware of the disease, with the majority of sufferers potentially not recognizing the symptoms [5], and the majority of COPD patients (80%) in Poland did not demonstrate a proper understanding of the disease. The low awareness of the study subjects also concerns smoking as the main risk factor of COPD. Smoking is the second greatest risk factor after arterial hypertension, and also the second most important factor contributing to DALY (*Disability Adjusted Life Years*) [6], COPD patients often appear to be not well informed about the close relationship between tobacco smoking and the development of the disease.

COPD is a disease which is not only treatable, but is also preventable. Many factors contribute to the occurrence of COPD, and most can be influenced by the patient. The main factor is tobacco smoking. It is responsible for 90% of cases [7], with COPD developing among approximately 20% of smokers [8]. The risk of COPD falls by half after quitting smoking [9]. However, it is estimated that 12.2% of never-smokers, aged 40 and above, also develop chronic obstructive pulmonary disease [10]. Unfortunately, smoking is still a very common habit among Polish citizens. In 2014, every fourth person admitted to daily smoking (30% of males, 21% of females); however, the number of smokers in Poland has fallen by 9% among males and 4% among females from 2003 to 2013 [11].

Together with smoking, the problems associated with an aging population represent a growing challenge for European countries. With age, the risk of developing chronic obstructive pulmonary disease increases. Even among never-smokers, the risk is more than 12-fold higher among people aged 80 years or above than among those aged 40–49 years [10]. It is estimated that the population of people aged 65 or older will increase in Poland from 13.5% in 2010 to 27% in 2030 [12], and this will be accompanied by a rise in the incidence of COPD, which is connected with the age of the population. An analysis of two key risk factors reveals that the aging of the population has a more significant effect on the incidence of COPD than tobacco smoking. This fact is corroborated by analyses and estimates conducted in the Netherlands. It was found that even if all persons living in that country had given up smoking in 1995 and no one had started to smoke, the percentage of people suffering from COPD would still have a growing tendency until 2015 [13].

Working and living in areas with high levels of air pollution and staying in poorly-ventilated rooms heated by fossil fuels are significant risk factors, and people with low socioeconomic status tend to demonstrate a greater risk of developing COPD [14]. COPD is also influenced by genetic factors, such as α-antitrypsin deficiency, and factors responsible for the development of the lungs in fetal life and in childhood (low birth weight). The disease is more likely to affect males rather than females [15]; however, in many countries, the male death rate due to COPD has been decreasing whereas the female death rate has been increasing [16].

The incidence of COPD is closely connected with the age of the population. While it is typically diagnosed in people over the age of 40, its onset occurs much earlier. In the adult populations of Europe, North America and Australia, the incidence of COPD ranges from 4 to 10% [17], and is predicted to continue rising in European countries until at least the year 2030 [18]. Few studies have examined the incidence of COPD in Poland, and the available data varies considerably, mainly due to the age of the studied populations; however, the most recent nationwide study found 20% of patients aged 60 years or older in the selected health care centers to be affected by COPD [19].

COPD is a significant social burden, and it contributes to a considerable number of hospitalizations, sickness-related absenteeism and disability. It was found to be the eleventh highest factor contributing to the value of DALY in 2002, and is expected to rise to the seventh position by 2030 [20]. It should be pointed out that it is the most common cause of mortality in a group of respiratory system diseases, which are the fourth most common cause of deaths in Poland after cardiovascular diseases, cancers, and accidents [21].

The number of deaths caused by a particular disease is closely associated with the number of years of lost life, reflecting the health burden placed on society. This study analyzes the years of life lost due to COPD during the period of 1999 to 2014 among the population of Poland and their change during that time.

## Material and methods

The data for the study comprises information gathered from 5,982,983 death certificates, owned by the Central Statistical Office in Poland. The database used in this article is available as supporting information (S1 File). The deaths occurred in Poland between 1999 and 2014. Poland has a 100% completeness of death registration. The authors made a detailed analysis of 110,217 deaths due to COPD, registered in the database as the underlying cause of death identified in the death certificate with the codes J40-J44 and J47, according to the three-digit codes specified by the International Statistical Classification of Diseases and Related Health Problems. The gathered information allowed the deaths to be analyzed by sex and place of residence, i.e. rural and urban areas, identified with territorial codes given by the Central Statistical Office [22]. Data regarding the size of the population was also received from the Central Statistical Office [23]. The SEYLL value due to COPD was calculated for each year of the studied period from 1999 to 2014.

The SEYLL index is considered a measure of premature mortality and a component of DALY, a measure of health identified as the total number of years lost due to mortality, which has been widely used for some time. The value is calculated according to Marshall [24], using the following formula:

$$SEYLL=\sum_{a=0}^{k}d_{a}e_{a}$$

where: *a*–age at which the person died,

*k*–age at which the oldest person in the studied population died,

*d_a_*–number of deaths of people at age *a*,

*e_a_*–number of expected years of life that remain to be lived by a person at age *a*,

In the study, the number of expected years of life was calculated using life expectancy values from the GBD 2010 Study [25]. In addition to the basic SEYLL index, both the SEYLL_p_ (per 10,000 people) and SEYLL_d_ (per single death) caused by COPD were calculated.

Changes in values of the above indices in the period 1999–2014 were evaluated with the use of the jointpoint regression model, which is an advanced version of linear regression,

y = a+bx,

where: y = ln(*z*),

*x*–calendar year,

and *z* is a measure evaluated in the study (SEYLL, SEYLL_p_ or SEYLL_d_).

Time trends were determined with the use of segments joining in joinpoints, where trend values significantly changed. To confirm whether the changes were statistically significant, the Monte Carlo Permutation method was applied. In addition, the authors also calculated APC (*Annual Percentage Change*) for each segment on the basis of generalized regression models and the Poisson distribution [26].

The AAPC (*Average Annual Percentage Change*) for the whole study period was also calculated [27]. Negative APC and AAPC values indicated positive changes in the analyzed measures, i.e.–their decreased value indicated diminishing incidence of chronic obstructive pulmonary disease, whereas positive values indicated negative changes–increased values of the analyzed measures indicated an increased incidence of chronic obstructive pulmonary disease.

In order to evaluate the statistical significance of APC and AAPC, the corresponding 95% confidence intervals (CI values) were also calculated. In all analyses, p≤0.05 was considered statistically significant.

The Joinpoint Regression program version 4.1.1.1 was used to analyze the time trends of the studied measures and to calculate the APC and AAPC parameters [28].

Changes in SEYLL, SEYLL_p_ and SEYLL_d_ values divided by sex and by place of residence (urban or rural) were evaluated with the application of the following criteria: males/females, urban/rural area.

The Bioethics Committee of the Medical University of Lodz (Poland) gave consent for the study to be conducted (no. RNN/422/12/KB).

## Results

COPD was found to contribute to 1.8% of the total number of deaths that occurred in Poland in the period 1999–2014. The percentage was the highest (2.1%) between 2007 and 2009 and the lowest (1.6%) in 1999 and 2014. Throughout the whole study period, COPD contributed to the majority of deaths attributed to chronic diseases of the lower respiratory tract (J40-J47): representing 80.6% of these deaths at the beginning of the analyzed period and 93.4% of them at the end. COPD was found to cause 34.3% of deaths due to diseases of the respiratory system (J00-J99) in 1999 and 29.5% in 2014.

The distribution of deaths due to COPD was almost equal between urban and rural areas; 50.4% if deaths took place in urban areas, and 49.6% in rural areas. In both areas, most COPD deaths were of males: 63.4% in urban areas and 77.4% in rural areas. In total, in the analyzed period, COPD contributed to 77,518 male deaths, constituting 70.3% of COPD-related deaths. Those who died of COPD were mostly middle-aged or older. Of the total number of COPD-related deaths which occurred in Poland in the period 1999 to 2014, less than 0.2% concerned those under the age of 40, irrespective of sex and place of residence. In Poland in the period under study, there was a considerable increase in the age group 80 and more in the share of deaths due to COPD (Table 1).

**Table 1:** Deaths due to COPD by sex and age in Poland in 1999 and 2014.

Age	Males				Females				Total			
	1999		2014		1999		2014		1999		2014	
	n	%	n	%	n	%	n	%	n	%	n	%
0–4	2	0.0	0	0.0	2	0.1	0	0.0	4	0.1	0	0.0
5–9	0	0.0	0	0.0	0	0.0	0	0.0	0	0.0	0	0.0
10–14	0	0.0	0	0.0	0	0.0	0	0.0	0	0.0	0	0.0
15–19	1	0.0	0	0.0	0	0.0	0	0.0	1	0.0	0	0.0
20–24	0	0.0	0	0.0	0	0.0	0	0.0	0	0.0	0	0.0
25–29	0	0.0	0	0.0	2	0.1	0	0.0	2	0.0	0	0.0
30–34	2	0.0	0	0.0	0	0.0	0	0.0	2	0.0	0	0.0
35–39	7	0.2	4	0.1	0	0.0	0	0.0	7	0.1	4	0.1
40–44	24	0.5	9	0.2	11	0.6	5	0.2	35	0.6	14	0.2
45–49	36	0.8	21	0.5	20	1.2	6	0.3	56	0.9	27	0.4
50–54	95	2.1	42	1.0	24	1.4	20	1.0	119	1.9	62	1.0
55–59	163	3.7	185	4.6	53	3.1	83	4.1	216	3.5	268	4.5
60–64	388	8.7	334	8.3	106	6.2	141	7.0	494	8.0	475	7.9
65–69	802	18.0	465	11.6	204	11.9	263	13.1	1006	16.3	728	12.1
70–74	1047	23.6	515	12.9	306	17.9	265	13.2	1353	22.0	780	13.0
75–79	922	20.7	770	19.2	315	18.4	299	14.9	1237	20.1	1069	17.8
80–84	473	10.6	892	22.3	253	14.8	421	21.0	726	11.8	1313	21.8
85+	483	10.9	770	19.2	412	24.1	501	25.0	895	14.5	1271	21.1
Total	4445	100.0	4007	100.0	1708	100.0	2004	100.0	6153	100.0	6011	100.0

The mean age of males who died of COPD in 1999 was 72.6 years (72.2 in cities and 72.9 in rural areas), while the mean age of females was 75.9 years (74.5 in cities and 77.8 in rural areas) at the beginning of the studied period and 76.8 years (76.3 in cities and 77.6 in rural areas) at the end.

Over the period 1999–2014, SEYLL values due to COPD changed dramatically. The most considerable decline (-31.8%) was noted in male inhabitants of rural areas, while an alarming rise (+27.2%) was observed in female urban residents. For both sexes, the urban/rural SEYLL ratio increased to 1.0 for males and 2.1 for females by the end of the studied period, indicating that the existing disproportion in the female group increased, and female inhabitants of cities still remained in a negative situation. In each analyzed place of residence, the SEYLL index was much higher in the male group, and although declining tendencies were observed in the whole analyzed period, the value of the male/female SEYLL ratio remained high.

**Fig 1:**
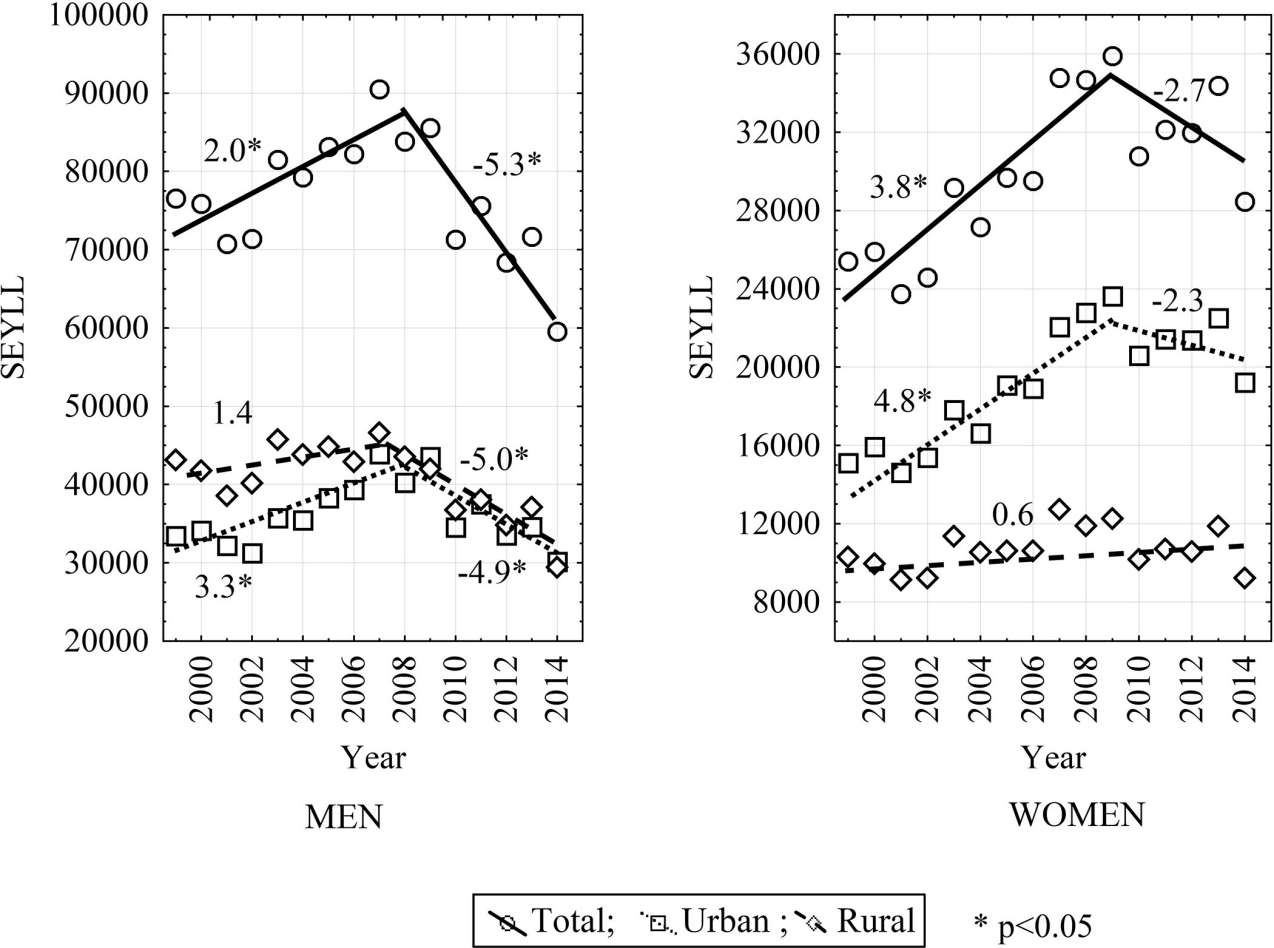
SEYLL trends by sex and place of residence in Poland from 1999 to 2014.

In 2014, the highest value (3.2) was noted among inhabitants of rural areas. An analysis of time trends revealed the greatest significant decrease in the value of the SEYLL index to occur among males in the period 2008–2014 (APC = -5.3; CI: -8.2 to -2.3; p<0.05). In contrast, the greatest increase in the value of the SEYLL index was observed among female urban residents over the period 1999–2009 (APC = 4.8; CI: 3.2 to 6.4; p<0.05). Female urban residents also demonstrated an alarming increase in the average annual percentage change of the SEYLL index (AAPC = 2.4; CI: 0.7 to 4.0). The only studied group in which the trend did not change significantly and remained almost the same over the studied period were the female rural inhabitants (APC = AAPC = 0.6; p>0.05) (Table 2). Fig 1 presents an analysis of the SEYLL index over time.

**Table 2:** Trends of standard expected years of life lost due to COPD by sex and place of residence in Poland from 1999 to 2014 with points of trend change and periods of decrease or increase in the SEYLL value identified in the Joinpoint Regression software.

Place of residence	Males							Females							Males/Females SEYLL ratio	
	SEYLL			Trend				SEYLL			Trend					
	1999	2014	% change	Period	AAPC	APC	95%CI	1999	2014	% change	Period	AAPC	APC	95%CI	1999	2014
Urban	33447.0	30134.2	-9.9%	1999–2014	-1.6		-2.6;1.5	15113.7	19232.0	+27.2%	1999–2014	2.4*		0.7;4.0	2.2	1.6
				1999–2008		3.3*	1.3;5.2				1999–2009		4.8*	3.2;6.4		
				2008–2014		-4.9*	-8.1;-1.5				2009–2014		-2.3	-6.6;2.1		
Rural	43122.7	29397.1	-31.8%	1999–2014	-1.6*		-3.0;-0.2	10293.2	9224.6	-10.4%	1999–2014	0.6	0.6	-0.6;1.8	4.2	3.2
				1999–2007		1.4	-0.7;3.5									
				2007–2014		-5.0*	-7.3;-2.6									
Total	76569.8	59531.2	-22.2%	1999–2014	-1.0		-2.4;0.4	25407.0	28456.6	+12.0%	1999–2014	1.6		-0.2;3.4	3.0	2.1
				1999–2008		2.0*	0.3;3.7				1999–2009		3.8*	2.1;5.6		
				2008–2014		-5.3*	-8.2;-2.3				2009–2014		-2.7	-7.3;2.1		
Urban/Rural SEYLL ratio	0.8	1.0	-	-	-		-	1.5	2.1	-	-	-		-	-	-

* p<0.05

In the period 1999–2014, the number of standard expected years of life lost due to COPD as calculated per 10,000 population, also changed significantly. In the analyzed period, the most significant decline was observed among males inhabiting rural areas (-34.9%) and the most significant increase was noted among females inhabiting cities (+29.3%). The above mentioned changes in the SEYLL_p_ index were due to its annual average 2% decrease among male rural inhabitants (AAPC = -2.0; CI: -3.4 to -0.6; p<0.05) and 2.4% increase (AAPC = 2.4; CI: 0.8 to 4.1; p<0.05) among female urban inhabitants.

The greatest annual percentage change in SEYLL_p_ was observed in the period 1999–2009 among female city inhabitants (APC = 4.9; CI: 3.3 to 6.5; p<0.05), and the greatest decrease in the period 2007–2014 among male inhabitants of rural areas (APC = -5.5; CI: -7.8 to -3.1; p<0.05) and in the period 2008–2014 among male urban inhabitants (APC = -4.9; CI: -8.2 to -1.4; p<0.05) (Table 3).

**Table 3:** Trends of standard expected years of life lost due to COPD per 10 000 population by sex and place of residence in Poland from 1999 to 2014 with points of trend change and periods of decrease or increase in the SEYLL_p_ value identified in the Joinpoint Regression software.

Place of residence	Males							Females							Males/Females SEYLL_p_ ratio	
	SEYLL_p_			Trend				SEYLL_p_			Trend					
	1999	2014	% change	Period	AAPC	APC	95%CI	1999	2014	% change	Period			95%CI	1999	2014
												AAPC	APC			
Urban	29.7	27.4	-7.7%	1999–2014	0.1		1.5;1.7	12.2	15.8	+29.3%	1999–2014	2.4*		0.8;4.1	2.4	1.7
				1999–2008		3.5*	1.5;5.5				1999–2009		4.9*	3.3;6.5		
				2008–2014		-4.9*	-8.2;-1.4				2009–2014		-2.3	-6.6;2.2		
Rural	59.3	38.6	-34.9%	1999–2014	-2.0*		-3.4;-0.6	14.1	12.1	-10.4%	1999–2014	0.3	0.3	-0.9;1.5	4.2	3.2
				1999–2007		1.2	-0.8;3.3									
				2007–2014		-5.5*	-7.8;-3.1									
Total	41.3	32.0	-20.1%	1999–2014	-1.1		-2.5;0.4	12.9	14.3	+11.0%	1999–2014	1.5		-0.2;3.3	3.2	2.2
				1999–2008		2.0*	0.3;3.8				1999–2009		3.8*	2.0;5.5		
				2008–2014		-5.5*	-8.4; -2.5				2009–2014		-2.8	-7.4;1.9		
Urban/Rural SEYLL_p_ ratio	0.5	0.7	-	-	-		-	0.8	1.3	-	-		-	-	-	-

* p<0.05

Similarly to the SEYLL index, the SEYLL_p_ index was much higher for males than for females over the analyzed period. These disproportions are reflected by the male/female SEYLL_p_ ratio values. Despite a decrease in the index value in the period 1999–2014, the males/females ratio was higher for rural inhabitants than for urban inhabitants. The male/female SEYLL_p_ ratio for 2014 was 1.7 for urban residents and 3.2 for rural residents.

**Fig 2:**
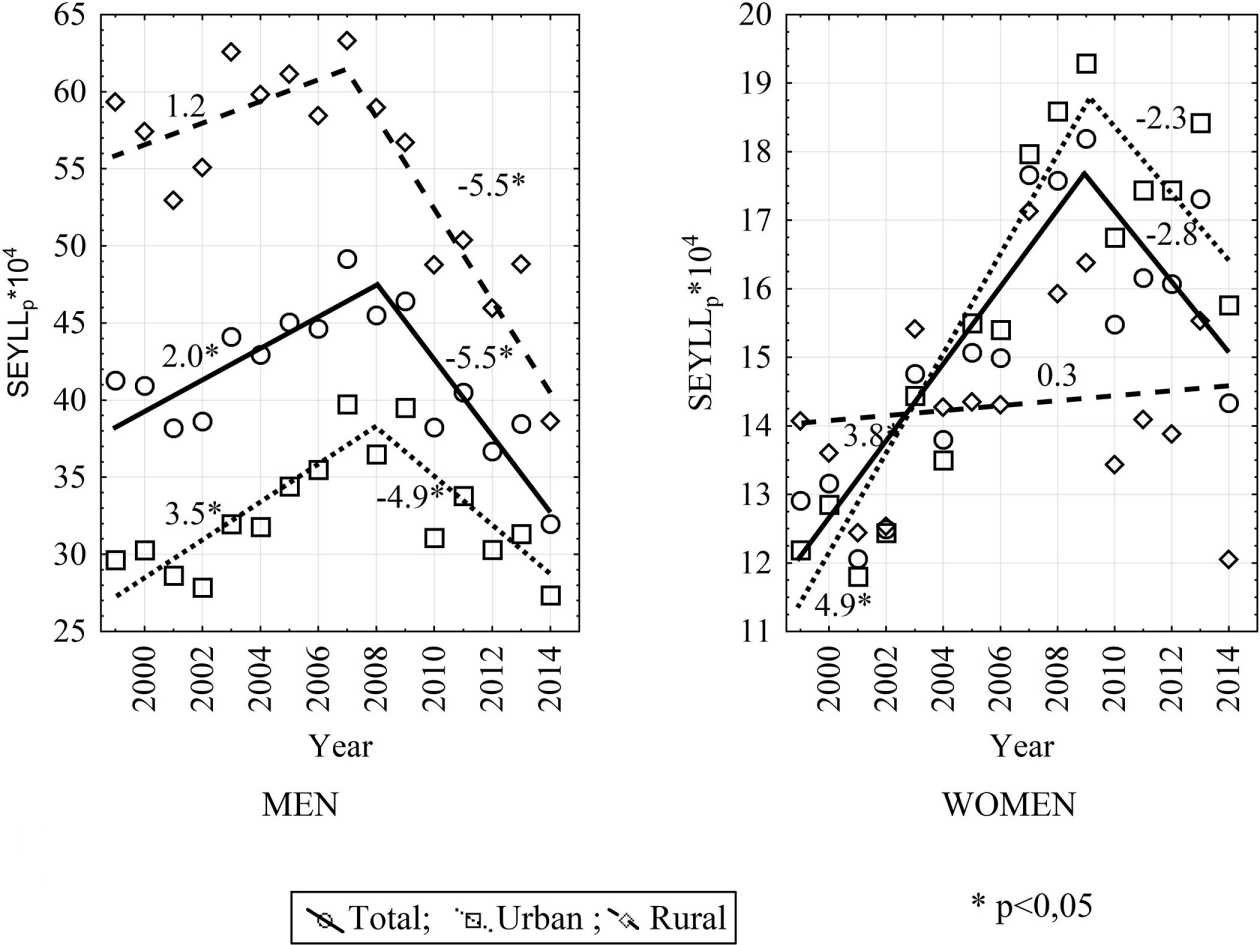
SEYLL_p_ trends by sex and place of residence in Poland from 1999 to 2014.

In the period under study, SEYLL_p_ values regardless of sex were higher for urban inhabitants than for rural inhabitants. This is shown by the urban/rural SEYLL_p_ ratio, which is below 1 in 1999 for males and females and in 2014 for males. Only among females in 2014 there was a higher SEYLL_p_ among urban inhabitants than rural inhabitants. Changing trends in the SEYLL_p_ index are given in Fig 2.

**Fig 3:**
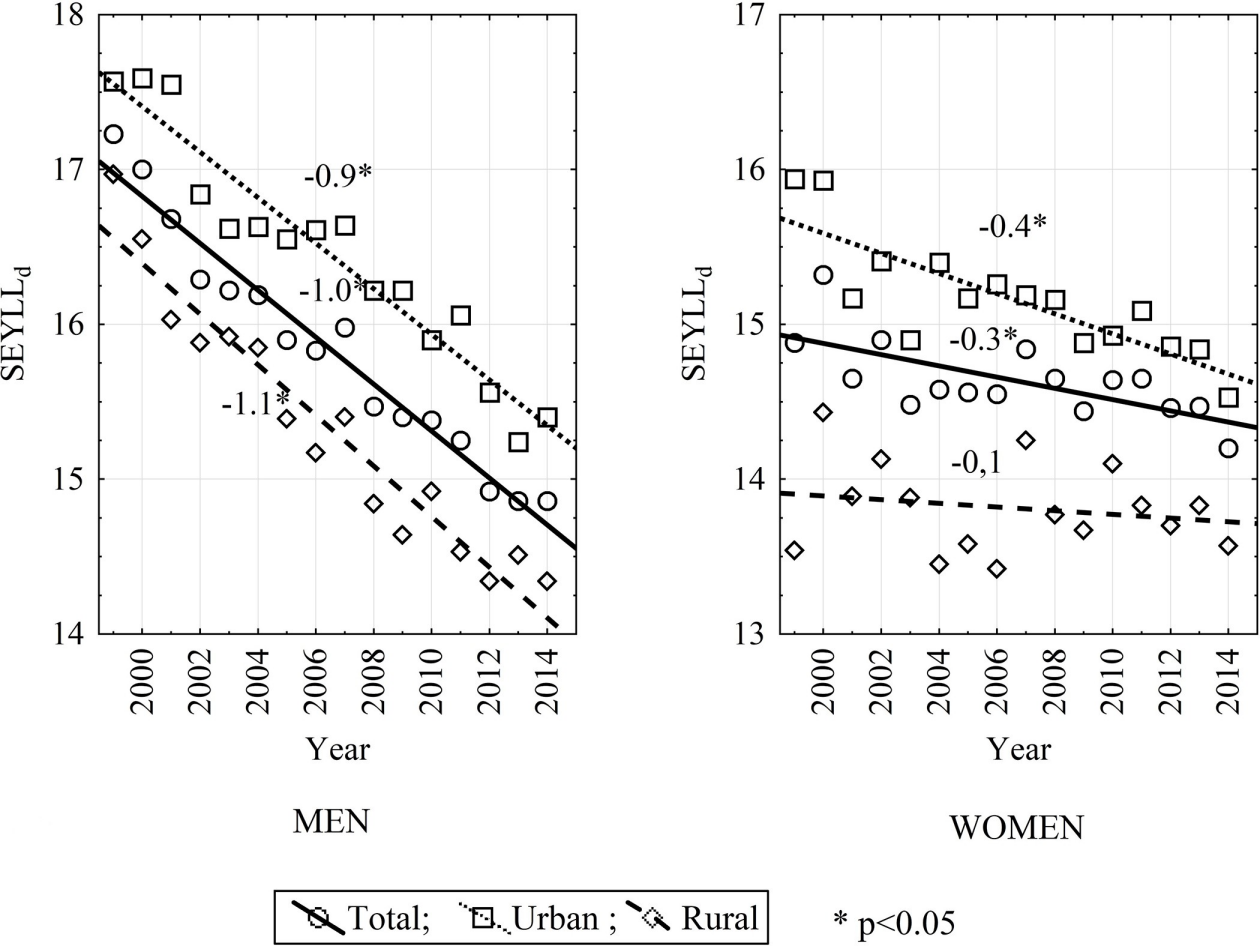
SEYLL_d_ trends by sex and place of residence in Poland from 1999 to 2014.

Of all analyzed measures, the slightest changes were observed in the value of the SEYLL_d_ index, i.e. the SEYLL calculated per individual person who died due to COPD. However, it should be pointed out that positive decrease tendencies were observed in that period: -15.5% among males living in rural areas, -8.8% among females living in city areas. Both the male/female SEYLL_d_ ratio and the urban/rural SEYLL_d_ ratio considerably changed over the studied years, with the values ranging from 1.0 to 1.3 in 1999 and 2014. Only the SEYLL_d_ index showed a negative trend. The most positive changes in the value of SEYLL_d_ was noted in the group of males living in the countryside (AAPC = APC = -1.1; -1.2 to -0.9; p<0.05) (Table 4), as given in Fig 3.

**Table 4:** Trends of standard expected years of life lost due to COPD per 1 death due to COPD by sex and place of residence in Poland from 1999 to 2014 with points of trend change and periods of decrease or increase in the SEYLL_d_ value identified in the Joinpoint Regression software.

Place of residence	Males						Females						Males/Females SEYLL_d_ ratio	
	SEYLL_d_			Trend			SEYLL_d_			Trend				
	1999	2014	% change	Period	APC	95%CI	1999	2014	% change	Period	APC	95%CI	1999	2014
Urban	17.6	15.4	-12.4%	1999–2014	-0.9*	-1.0;-0.7	15.9	14.5	-8.8%	1999–2014	-0.4*	-0.6;-0.3	1.1	1.1
Rural	17.0	14.3	-15.5%	1999–2014	-1.1*	-1.2;-0.9	13.5	13.6	+0.2%	1999–2014	-0.1	-0.3;0.2	1.3	1.1
Total	17.2	14.9	-14.0%	1999–2014	-1.0*	-1.1;-0.8	14.9	14.2	-4.6%	1999–2014	-0.3*	-0.4;-0.1	1.2	1.0
Urban/Rural SEYLL_d_ ratio	1.0	1.1	-	-	-	-	1.2	1.1	-	-	-	-	-	-

* p<0.05

## Discussion

### Review of principal own results

Our study on years of life lost due to premature mortality caused by COPD with the use of the following measures: SEYLL, SEYLL_p_ and SEYLL_d_ is the first one in the population of Poland. We were inspired to undertake the detailed analysis by the growth in the percentage share of deaths caused by disorders of the respiratory system (J00-J99 according to ICD-10) in the period under study from 4.70% (the absolute number was 17,943) in 1999 to 5.41% (20,378) in 2014 in the total number of deaths in Poland [29].

Respiratory system diseases ranked fourth in the hierarchy of mortality in Poland, following cardiovascular diseases, malignant neoplasms and deaths due to external causes. In the period under study, there was a small change in the number of deaths due to COPD (J40-J44 and J47 according to ICD-10). In 1999, there were 6,153 deaths from this cause, while in 2014–6,011 deaths.

During the period of 16 years under study, positive changes regarding years of life lost due to COPD were observed in Poland among males. The SEYLL in 1999 amounted to 76,569.8 years and declined by 22.2%, reaching the value of 59,531.2 years in 2014. A higher dynamic of the reduction in SEYLL (31.8%) was observed among males living in the rural areas compared to males in the urban areas, where the SEYLL reduction was only 0.9%. A worrying situation of an increase in the years of life lost due to COPD was observed among females living in Polish cities, as the SEYLL rose from 15,113.7 years in 1999 to 19,232.1 in 2014, i.e. by 27.2%. This contributed to the unfavorable growing trend by 12.0% of years of life lost due to COPD in the whole population of Polish females (from 25,407.0 to 28,456.6 years) in spite of a 10.4% reduction in SEYLL_p_ due to this cause among women living in the rural areas. Analyzing the trend of changes with the use of SEYLL_p_ per 10,000 population, two phases are visible. During the first decade of our research (1999–2008) there was a growth, and subsequently, a reduction in this indicator in the period of 2008–2014 with the exception of SEYLL_p_ for women living in the urban areas, where there was an increase in the whole period under study.

During the whole period under study, the analyzed indicator was higher among males than females. In 2014, it amounted to 32.0 per 10,000 males and 14.3 per 10,000 females. It is worth noting that differences in the value of SEYLL_p_ between both sexes were observed for all respiratory system diseases: in 2014, 43.5 per 10,000 males and 22.0 per 10,000 females [30].

This may be due to the prevalence of tobacco smoking among males and females as well as the exposure to tobacco smoke and other risk factors [31]. Smoking as a very important risk factor for COPD incidence continues to be frequent in the population of Poland. In 2014, every fourth adult admitted smoking every day. Males smoked more often (30%) than females (21%). The recently noticeable decrease in the share of smoking Poles was weaker for females [11, 32]. Furthermore, the higher reduction in SEYLL_p_ in the rural areas than in cities may be related to a worse quality of air in the urban areas.

It needs to be underlined that positive changes in the decline of the SEYLL_d_ indicator were observed among both sexes and in both living environments. However, a male who died of COPD in Poland in 2014 lost on average 14.9 years of life, while a female– 14.2 years. For the sake of comparison, SEYLL_d_ due to bronchial asthma amounted to 19.8 years for males and 15.3 years for females in Poland in 2013 [33]. An improvement in this indicator may be expected thanks to better recommendations for prevention and pharmacological and non-pharmacological treatment [34].

### Results of other studies on COPD mortality

Epidemiological data on COPD prevalence and mortality differs considerably among countries. In 2007, the prevalence of COPD in Europe ranged from 2.1% to 26.1% depending on the age of the study subjects. The standardized mortality rates due to COPD by age ranged from 7.2 in France to 36.1 in Hungary per 100,000 inhabitants. In Poland, they amounted to 16.8 overall, 32.0 for males and 7.8 for females [35]. Over the period 1994 to 2010, the standardized death rates (SDRs) due to COPD in Poland fell insignificantly by 0.3% per year for males but increased significantly by 1.6% per year for females. These changes appeared to be positive in comparison to the mean value for the 27 European Union countries [36]. This variation results from the use of different COPD detection criteria, different methods of conducted studies and the age of the analyzed populations [37]. Only the implementation of GOLD (*Global Initiative for Chronic Obstructive Lung Disease*) and PLATINO (*Latin American Project for Investigation of Obstructive Lung Disease*) criteria allowed more coherent sets of results to be obtained [38, 39].

According to the GBD Study conducted in 2015, COPD caused 3.2 million deaths globally, which means an increase in 11.6% compared to 1990. The number of years of life lost due to COPD in 2015 amounted to 51,803 thousand, whereas the number of years of life with disability amounted to 12,047 thousand, causing 63,850 thousand DALYs. Years of life lost (YLL) contributed to over 80% of DALYs due to COPD. Poland belongs to a group of countries for which the age-standardized DALY rate per 100,000 people is situated in the interval of 301 to 600 [40].

### Limitations of the study

The study had several limitations.

First of all, the death cause is established on the basis of information about the initial cause of death written in the death certificate by the doctor stating death. The credibility of this data in Poland is questioned. It is estimated that deaths referred to as the so-called “garbage codes” accounted for over 25% of all deaths in Poland in 2012. It may be suspected that there is an underestimation of deaths due to COPD.

In Poland in 2009, changes were introduced aiming at improving the quality of data on death causes. The doctor putting the death cause into the death certificate is responsible for this statement. Moreover, the information on the death cause according to the ICD-10 classification is subject to control and verification by qualified teams of coders.

Another limitation of our study is the 16-year-long observation period, which may be too short to establish significant trends in the values of the analyzed indices. The too short period of observation is particularly important in the context of the different quality of data on death causes before 2009.

It is worth pointing out that COPD is accompanied by many concomitant diseases including cardiovascular diseases (myocardial infarct, cerebral stroke, atherosclerosis, pulmonary embolism), osteoporosis, anemia, skeletal muscle atrophy, diabetes, metabolic syndrome and depression. Lung cancer is also commonly observed [41]. Most diseases which are concomitant with COPD significantly affect the statistics, particularly those regarding mortality; for example, cardiovascular diseases contribute to more than 25% of all deaths of patients diagnosed with COPD [42]. As these concomitant diseases are frequently an underlying contributor to death, they lead to an underestimation of data on COPD mortality [43].

Moreover, YLL studies use various indicators and various methods of their calculation. An example of it may be SEYLL analysis for the mortality of Poles in 2011 conducted by the authors of this paper. It showed that the group of deaths coded as J40-J47 in ICD-10, i.e. a wider group than in the current study, represented the 11^th^ highest cause of death for men and the 15^th^ highest for women with regard to the SEYLL_p_ index. These positions were 15^th^ for men and 14^th^ for women with regard to SEYLL_d_ [44]. Chronic diseases of the lower respiratory tract were in a much higher position than cardiovascular diseases. The values regarding years of life lost obtained by the present analysis differ from those obtained in 2011, as current analyses are based on GBD guidelines based on the table of standard expected years of life recommended by Murray et al. [25], without age weighting or discounting. It is assumed that values regarding years of life lost, undiscounted and unweighted, are approximately half higher than indices discounted by time and weighted by age [45].

### The importance of fighting COPD as an element of the health policy

Our analyses clearly indicate that COPD poses a serious problem in the Polish population, and that worse results were observed in the male group. A huge gap exists between the results for males and females, despite a significant decrease being observed in standard expected years of life lost among males and a simultaneous increase, or a slight decrease, in this parameter among females. They also show a growing difference between rural and urban inhabitants.

COPD represents a health burden for Poles and a great challenge for public health in terms of reducing its incidence and improving the effectiveness of diagnostics and therapeutics. In order to lessen the problem of COPD, it is important to detect the disease early and reduce levels of air pollution. As the Polish population is aging rapidly, the first step is to eliminate modifiable risk factors. Such elimination requires the implementation of many educational projects, aimed at familiarizing the population with the negative impact of smoking and increasing their health awareness. These steps will successfully eliminate health inequalities associated with place of residence and sex.

## Supporting information

S1 File This is the database used in this article. (XLSX)

## Abbreviations

AAPC: average annual percentage change
APC: annual percentage change
CI: Confidence Intervals
COPD: chronic obstructive pulmonary disease (J40 –J44 and J47 according to ICD-10)
DALY: Disability Adjusted Life Years
GOLD: Global Initiative for Chronic Obstructive Lung Disease
PLATINO: Latin American Project for Investigation of Obstructive Lung Disease
SEYLL: Standard Expected Years of Life Lost
SEYLL_p_: Standard Expected Years of Life Lost considering living population
SEYLL_d_: Standard Expected Years of Life Lost considering number of deaths.
